# Plausible Emergence of Autocatalytic Cycles under Prebiotic Conditions

**DOI:** 10.3390/life9020033

**Published:** 2019-04-04

**Authors:** Stefano Piotto, Lucia Sessa, Andrea Piotto, Anna Maria Nardiello, Simona Concilio

**Affiliations:** 1 Department of Pharmacy, University of Salerno, Via Giovanni Paolo II, 132, 84084 Fisciano SA, Italy; lucsessa@unisa.it (L.S.); apiotto@hotmail.com (A.P.); annardiello@unisa.it (A.M.N.); 2 Department of Industrial Engineering, University of Salerno, Via Giovanni Paolo II, 132, 84084 Fisciano SA, Italy; sconcilio@unisa.it

**Keywords:** origin of life, hypercycle, Monte Carlo

## Abstract

The emergence of life in a prebiotic world is an enormous scientific question of paramount philosophical importance. Even when life (in any sense we can define it) can be observed and replicated in the laboratory, it is only an indication of one possible pathway for life emergence, and is by no means be a demonstration of how life really emerged. The best we can hope for is to indicate plausible chemical–physical conditions and mechanisms that might lead to self-organizing and autopoietic systems. Here we present a stochastic simulation, based on chemical reactions already observed in prebiotic environments, that might help in the design of new experiments. We will show how the definition of simple rules for the synthesis of random peptides may lead to the appearance of networks of autocatalytic cycles and the emergence of memory.

## 1. Introduction

The realization of a unified concept of life, incorporating all known biological systems separated from physical and chemical systems, is a long and complex process that is far from conclusion. Addy Pross [1] argues that the question about how life emerges is strongly connected with the definition of life. According to the synthetic theory of evolution, “life is a self-sustained chemical system capable of undergoing Darwinian evolution” [2]. This definition can be considered a derivation of another definition, formulated by M. Perret and elaborated upon by J. D. Bernal [3], that states: “Life is a potentially self-perpetuating system of linked organic reactions, catalyzed stepwise and almost isothermally by complex and specific organic catalysts which are themselves produced by the system.”

A persuasive model of the origin and evolution of life came from the work of Eigen and Schuster in 1977. In their work [4] they suggested that self-replicative macromolecules, such as RNA and DNA in a suitable environment, exhibit behavior which we may call “Darwinian” and which can be formally represented by the concept of the “quasi-species.” Though sophisticated, the hypercycle model requires other requisites to realize the condition of life. Compartmentalization is necessary to prevent dilution of chemicals and the stability of hypercycles but does not take into account the emergence of homochirality and the memory of the system [5].

The present work originates in this theoretical framework. We aim to address the problem of the emergence of a stable order in the spontaneous and prebiotic synthesis of peptides.

A system is considered “alive” when it is capable of producing its constitutive elements, though with some errors, and when it is capable of replicating itself, keeping roughly the same composition. These conditions define a system as autopoietic. The synthesis of constitutive materials is a necessary, though not sufficient, condition. It is essential to propose a way in which a system could keep the memory of its composition.

In living beings, the information about what must be synthesized, and when, is stored in DNA or RNA molecules. The genetic code is the map that translates the codon into an amino acid. In modern-day biology, this translation is accomplished by means of the ribosome, a complex molecular machine which was not available for the first emergent life. Let us assume that, before the appearance of a genetic code, it was necessary to have a system to maintain the memory of the formed molecules.

According to the RNA-world scenario [6], RNA molecules could provide both the information storage (in their sequences) and the catalytic activity. In the RNA scenario, proteins are not required in the early stage of life development. Nevertheless, the RNA-world was fiercely opposed [7] because of two orders of reasons: The prebiotic synthesis of RNA is very difficult, and RNA molecules catalyze only a few reactions, mainly the cleavage of RNA, despite the observation that all types of reactions necessary for nucleotide and peptide synthesis can be catalyzed by ribozymes [8]. RNA-world requires the prebiotic synthesis of some molecules, written information (in this case RNA molecules), and machinery to read and execute those data. The information must be copied and shared among the progeny.

In our opinion, the most severe critique of the RNA-world scenario is that the information can be stored in RNA molecules only with the help of a complex (protein) machinery. In more general terms, memory is hard to fit in any model without storage. 

Many research works demonstrated that amino acids could be formed in prebiotic times, along with other organic compounds. The famous Miller–Urey experiment, for example, produced a mixture of amino acids [9]. Several amino acids have been found even in meteorites [10]. A consensus list of prebiotic amino acids includes Ala, Asp, Glu, Gly, Ile, Leu, Pro, Ser, Thr, and Val [11]. The oligomerization of amino acids to form new peptides is extremely hard to predict.

Several experiments demonstrated that the presence of small peptides in early Earth was plausible [12]. Peptide growing and degradation was considered in the pioneering work of Kauffman [13] and Eigen [14], for a curious case in the same year. In 1971, Eigen proposed the hypercycle, based on Orgel-type replicating “Watson–Crick” RNA strands, but with the additional idea that “a replicating pair 1 would catalyze or help the replication of 2, 2 would help 3 and so on, until N closed the ‘hypercycle’ and helped replicate 1”.

Kauffman made the radical simple assumptions that the early molecules were polymers of two types of monomers, A and B, say two amino acids, or later, two nucleotides. These could undergo only cleavage and ligation reactions. Monomers A and B; and dimers AA, AB, BA, and BB, might serve as sustained food inputs to the system. Given this, the reaction network among the molecules could be determined. In 1971 the formation of medium or long peptides by amino acid polymerization was not yet wholly investigated experimentally. Many researchers share the idea that some random peptides could have been formed spontaneously and thus helped the onset of protobiological mechanisms (like autocatalytic and cross-catalytic cycles, complex formation with metals or other non-peptide molecules, membrane functionalization and control of trans-membrane traffic, new peptide formation by chain elongation or fragment condensation, synthesis and stabilization of nucleic acids, and so on) [15,16,17,18,19,20,21].

Since the work of Kaufmann and Eigen, several attempts have been made to simulate the emergence of autopoietic structures on a computer, but it very soon appeared clear that the number of required structures was too high to be calculated. A hundred amino acids can be combined in circa 10^130^ possibilities. The chemical space is too vast to be investigated either experimentally or computationally. The number of options is so vast that a particular sequence of 100 amino acids could likely have never appeared on our planet. Not only that, it must be considered that a single copy of a protein is not enough to sustain reproduction. It is important to have the accumulation of a particular protein for an organism to have an evolutionary advantage.

Nature could not have verified all possible amino acid combinations and, therefore, we face the problem of how the “few” existent proteins were produced and/or selected during prebiotic molecular evolution and, subsequently, how through a series of spontaneous steps of increasing molecular complexity they could produce life as self-reproducing protocells. Do existing proteins have some specific features that make them eligible for selection, for example in terms of particular thermodynamic, kinetic, or other physical properties? 

Let us assume a system similar to the one proposed by Urey in 1953 [22]. We can hypothesize the spontaneous synthesis of amino acids and the regular production of fresh material. Let us assume that some kind of polymerization can take place on a mineral surface (or clay [23]) to catalyze the elongation of short peptides. The presence of a surface offers the advantage of spatial confinement of the amino acids that may lead to high peptide concentrations. Let us assume a continuous formation and degradation of peptide taking place onto the surface. 

We can imagine the rain washing away the newly synthesized materials from the mineral surface, and the sun evaporating water and increasing the peptides’ concentrations in these ponds. At high concentrations, these peptides can interact with each other. Any theoretical model should consider the presence of competing chemicals or parasites. Parasites of hypercycles may interrupt the cooperation of replicators [24].

The study of the emergence of autocatalytic cycles from short peptides presents some other critical problems. The first one is related to the number of possible sequences that can be generated. The astronomically large number precludes the possibility of exhaustive analysis. The second problem is the identification of the onset of such a cycle. Instead of using the twenty natural amino acids, we decided to limit their number to those present in prebiotic conditions only. 

Within this theoretical framework, we aimed to evaluate the condition for the onset of hypercycles, while addressing the stability against parasite sequences and the establishment of a kind of dynamic memory. We have written a computer program (Genesys) to reproduce the chemical synthesis of random peptides. As starting pool, we chose the amino acids present in the Murchison meteorite [25] (A, V, G, E, D, and S) with concentrations inversely proportional to the formation energy, and a reservoir of amino acids to fish from. 

With these six amino acids in large quantities, we have defined some rules that, once set, should permit the emergence of new properties. We began to catalyze the first reactions among amino acids, and we observed the formation of dimers; these dimers can, in turn, lengthen or degrade and return to their initial state. If the speed of synthesis is slightly higher than the speed of degradation, gradually longer peptides will accumulate. Shorter peptides will be slightly more stable than longer ones because the latter can break into many pieces. Following this approach, after a few generations of synthesis and degradation, an enormous number of different sequences will appear. Some sequences could be “parasitic” and compete with emerging hypercycles. 

We have explored the possibility of exploiting the similarity among sequences to drastically reduce the number of synthesized peptides and to protect hypercycles from parasites. The similarity among peptides could favor the aggregation, similar to what happens in plaque formation. Even the simplest form of peptide activity, proteolysis, can direct the cut of an elongated peptide in a position that would lead to the accumulation of similar peptides.

Of course, a peptide α may interact with a peptide β that shares only a portion of the entire sequence, and still will lead to the accumulation of α or β, or more, peptides. The idea of similarity increasing the concentration of different peptides can be misleading. One may think that proteolysis would dramatically increase the number of possible sequences, but similarity can play a more sophisticated, and still spontaneous, role. For example, in the case of elongation, it may lead to the propagation of subsequences across the population. This step permits an increase in the number of available three-dimensional structures, and, at the same time, limitation of the number of sequences. Rather than having the accumulation of a myriad of different peptides, one can observe an increase in the structural complexity without an explosion of the number of peptides. With the onset of new kinds of sequences, we may find new three-dimensional structures, and new properties may emerge. 

As the number of possible combinations for analysis increases with the square power of the number of sequences, it is clear that this method would not lead to any peptide. It is well known that two similar sequences generally have similar three-dimensional structures and biological functions.

We assumed that similar peptides could more easily aggregate to very different peptides and, in this way, have a lower probability of degradation. This hypothesis favors the persistence of similar sequences but does not favor their development. To have an accumulation of a particular sequence, it is necessary to have a sort of “memory” of the system. In biological times, memory is offered by specialized molecules that preserve the information of the sequences (DNA or RNA), together with a code for their translation. In prebiotic times, memory can emerge dynamically with autocatalytic cycles exploiting the similarity between two sequences to favor a template effect. Let us imagine four peptides (A, B, X, Δ) as these reported:

A   GGGAAAVGVGAA

B   GGGAAAAGAGVVVVAAGVAV

X   AAVGVGAVVAAVVA

Δ   VAAGVAVGGGVGAA

We can hypothesize that, with the help of similarity-driven catalysis, a cycle of this type may emerge:


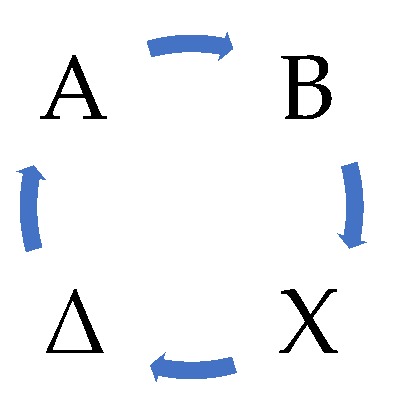


In the presence of autocatalytic cycles, the concentration of peptides {A, B, X, Δ} may vary cyclically. This means that the number of occurrences of particular sequences will not change in monotonous ways, but cyclically. More precisely, the periodic variation in concentration of a specific peptide is a necessary, but not sufficient, condition for the appearance of a cycle. It is important to note that we are considering the concentration oscillations arising from an autocatalytic activity, rather than fluctuation induced by external factors (temperature, water concentration, or lightning). 

The primary objective of the present work is to set up the conditions by which such an autocatalytic cycle can emerge spontaneously from the random synthesis of linear peptides. Here we want to test if the introduction of similarity may lead to the onset of autocatalytic cycles. The onset of self-sustaining autocatalytic cycles would represent a rudimental and primordial form of memory in the system without the need of DNA or RNA. 

## 2. Materials and Methods

Genesys is a program written in C++ that allows the user to build random libraries of peptides. From an initial set of amino acids, Genesys uses a Monte Carlo method to bind two amino acids or to lengthen and shorten the present peptides. At each generation, Genesys randomly chooses between present peptides and amino acids and proceeds with the operations of synthesis and fragmentation.

The calculation is iterative. Each “generation” consists of the following steps: starting pool, synthesis, and fragmentation. Starting pool represents the set of all the amino acids and all the starting oriented polypeptides. The method takes into account the orientation of the polypeptides. For example, the polypeptide GENESYS is different from the polypeptide SYSENEG because each sequence is oriented in the N-C direction. 

The synthesis starts randomly from the first molecule of the pool that is randomly coupled with another molecule. “Randomly” represents the analog of the immediate proximity of two molecules in the real world. Once the pairs are made, the algorithm proceeds to calculate the probability P that the two polypeptides can combine. Let us define two strings: S1 and S2. P is calculated in terms of their length and on the basis of their similarity (*simil*) as defined in the following:

(1) $$\begin{array}{c} \mathrm{If}\;(length\;S1\leq 3\;AND\;length\;S2\leq 3)\;\\ P=c1*c2\\ \mathrm{else}\\ P=c1*c2*simil \end{array}$$

Whereas

(2) $$simil=\frac{\max H}{\min\left(length\;S1;\;length\;S2\right)};\leq 1$$

Length S1 and S2 represent the number of amino acids constituting the two peptides of the pair; c1 and c2 are the interaction coefficients as arbitrarily defined in the program to favor the interaction of longer peptides (listed in Table 1). 

*MaxH* is the maximum of the substitution matrix *H.* The matrix is built following the same criteria of the BLOSUM matrix [26]. It is used to score alignments between different sequences. *H* is built by progressively finding the matrix elements, starting at *H**_1,1_*** and proceeding in the directions of increasing *i* and *j*. Each item is set according to:

(3) $$H_{i,j}=max\{\begin{array}{c} 0\\ H_{i-1,j-1}+\;S_{i,j}\\ H_{i-1,j}-d\\ H_{i,j-1}-d \end{array}$$

The *S_i,j_* is the similarity score of comparing two amino acids (obtained from the similarity Table 2 as defined in Equation (4)), and *d* is the penalty for a single gap. The penalty gap *d* is set to 2.

(4) $$S_{i,j}=\;\frac{10}{10+\left|S_{i}-S_{j}\right|}$$

The values of *S_i,j_* range from 0 to 1. The similarity table (Table 2) is derived from chemical–physical properties such as polarity and ΔG of solvation, where the polarity (polar) is taken from [27], and the amino acid energy of solvation ΔGw is taken from [28].

The similarity coefficients (S) are obtained by rounding the results of the following equation [28]:

(5) $$S=\;-69+3\cdot \mathrm{polar}+1.3\cdot \Delta \mathrm{Gw}+\;\sqrt[{3}]{-6.5\cdot \mathrm{polar}}+\;\sqrt[{3}]{-34.9\cdot \Delta \mathrm{Gw}}\;$$

The values range permits calculation of the similiarity between two sequences in a BLOSUM fashion. 

Once the synthesis is complete, the program moves to the fragmentation, with a probability P that is proportional to the length of the polypeptide.

(6) $$P=\frac{length}{\mathrm{threshold}};P\leq 1$$

The threshold is the length beyond which the polypeptide is certainly fragmented. Once fragmentation is complete, the set of new molecules represents the starting pool of the new generation.

We used as the starting pool the six amino acids (A, V, G, E, D, and S) found in the Yamato meteorite [29], for which plausible prebiotic synthesis pathways are known. The initial pool can be modified during the experiment to simulate the appearance of new molecules in the amino acid pool. 

The program generates text files containing the sequences and the number of occurrences in the sample. The similarity was calculated by multiple alignments with the ClustalW algorithm implemented in MEGA7 [30], with the Jones, Taylor, and Thornton (JTT) method [31]. Identical sequences have a distance of 0. A threshold for the distance values of 0.300 was set. Due to the large number of amino acid sequences obtained, they were clustered on the basis of their length and similarity in order to be able to analyze them. In this way, for two or more extremely similar sequences only one representative sequence was taken, increasing its number of occurrences. The clustering process is called epoch. The sequences obtained after clustering were submitted again to the program for a new epoch. 

The whole experiment saw the creation of 20 epochs, equal to 8000 generations. 

In the restart phase, equivalent to a second experiment, we wanted to verify that if a sequence was eliminated from one of the previously generated epochs, the one closely related to it would not have formed. We proceeded, therefore, with the elimination of a sequence from epoch 4, and we repeated the previously described protocol for the remaining epochs. The choice of the sequence to be eliminated is arbitrary.

## 3. Results

At the beginning of the simulations, peptides can catalyze only two processes: the lengthening of similar peptides and the shortening of peptides at precise positions. As a result, peptide building blocks begin to form. Kauffman hypercycles can emerge spontaneously if the system continues to grow in complexity without the formation of an astronomical amount of peptides. In fact, the number of sequences produced by the Monte Carlo method increases extremely rapidly and exponentially. After a few hundred generations, the number of peptides generated is in the order of 10^5^, and the number of those surviving the degradation is 10^3^.

It is, therefore, reasonable to cluster similar sequences and let only the representatives of each cluster of similar peptides in the sequence pool. The number of occurrences of these sequences represents the size of the cluster. Each group of similar sequences tends to fade over the generations due to the degradation of the peptides. To be sure that the presence of oscillations in the number of occurrences is neither an artifact of the algorithm nor a stochastic fluctuation, starting from epoch 4 we deleted some sequences. The choice of the peptide to be removed depends on their condition of variable and periodic concentration. It is important to remember that, in the course of the analysis, we indicate as sequence a cluster of n sequences with reciprocal distance lower than the threshold value. If the sequence is part of a Kauffman cycle, variations in the concentrations of the other sequences of the hypercycle should be observed. For very complex systems, with different interacting hypercycles, not even the deletion of one sequence would lead to the complete suppression of the other peptides of the cycle.

Following the suggestion of Eigen, we could hypothesize that the peptide A could catalyze the formation of B, that B catalyzes the formation of X, and the latter could catalyze the formation of A. In this simple cycle, each peptide catalyzes the formation of other peptides and ultimately sustains its creation. 

**Figure 1:**
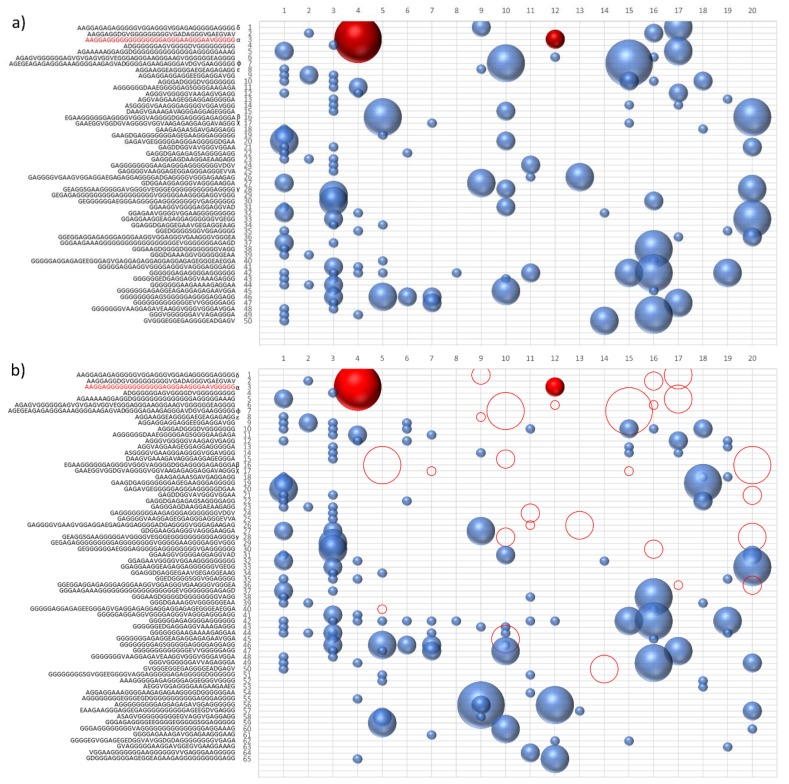
Clusters of similar sequences for each epoch. The number of copies of each group is shown as sphere size (**a**). Evolution of sequences after 8000 generations starting from a pool of six amino acids. (**b**) Evolution of sequences after the removal of the sequence in red from the pool at epoch 4 (corresponding to 1600 generations). Empty circles correspond to sequences that do not appear in the evolutive process upon the removal of the sequence in epoch 4.

As we can see in Figure 1, the ability of the similarity to increase the frequency of polymerization has led to the presence of numerous sequences for which the concentration is oscillating over the generations. To highlight the presence of the hypercycle we have set equal to zero the number of occurrences of the sequence α (see Table 3) AAGGAGGGGGGGGGGGGGAGGGAAGGGAAVGGGGG. Figure 1b shows the sequences that disappear in subsequent generations once the α sequence has been deleted. This result cannot be considered conclusive proof of the presence of interconnected cycles, but it suggests the high level of interconnection of the considered peptide system.

The system of peptides represented in Figure 1a,b suggest how different sequences are associated with sequence α. To confirm the presence of an autocatalytic cycle, we have eliminated, again at epoch 4, another sequence chosen from among those that appear to interact with the sequence α.

**Figure 2:**
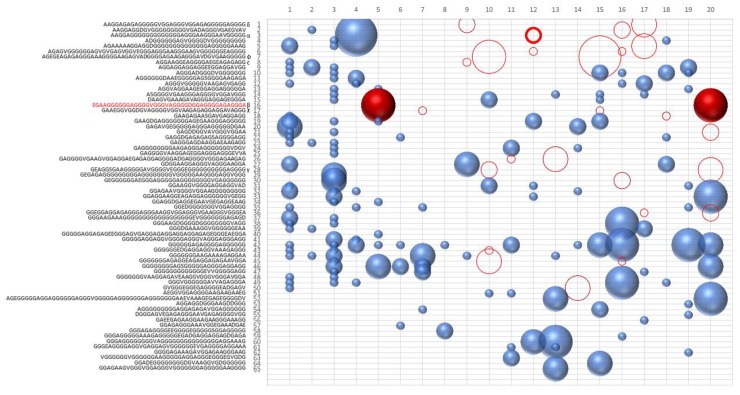
Clusters of similar sequences for each epoch. The number of copies of each cluster is shown as sphere size. Evolution of sequences after the removal of the sequence in red from the pool at epoch 4 (corresponding to 1600 generations). Empty circles correspond to sequences that do not appear in the evolutive process upon the removal of the sequence in epoch 4. For the sake of clarity, with the marked empty red circle, we indicate the sequence α.

Figure 2 shows the result of the new evolution.

**Figure 3:**
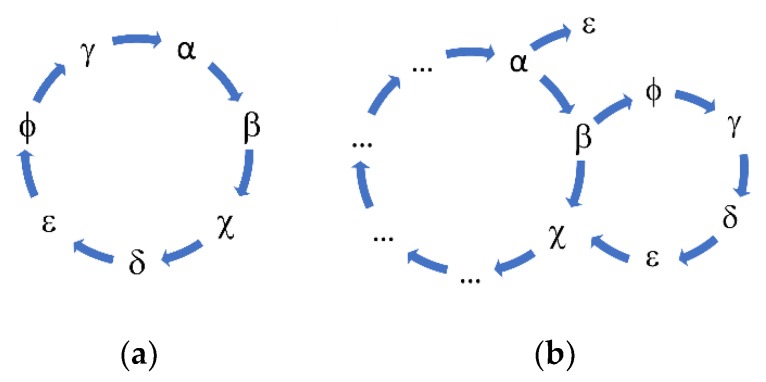
The hypercycle that emerged spontaneously imposing a similarity control to peptide replication. (**a**) An isolated hypercycle; (**b**) two interconnected hypercycles.

The deletion of the sequence β (see Table 3) causes the extinction of the sequences χ, δ, and ε. The sequences belonging to the same hypercycle are listed in Table 3. The deletion of the same sequences by α or β confirms that α and β belong to the same hypercycle. Figure 3 shows some representative sequences obtained in the described experiments.

The presence of self-catalytic cycles is a necessary condition for the emergence of life, but not a sufficient one. We have observed that longer sequences can be formed and how sequences can catalyze the formation of similar sequences and therefore the establishment of self-catalytic cycles.

The obtained results are not accidental. We have repeated the same experiment from 50 slightly different initial conditions varying the concentration of glycine in the initial pool. The pool was constituted by the main six amino acids with constant concentration of 38 A, 4 D, 11 E, 100 G, 1 S, and 10 V. In the validation set we span the number of glycine from 75 to 124 and, in every case, we have observed the emergence of hypercycles. The sequences tend to be different in the exact composition but similar as calculated with Equation (4). In the Supplementary Information we have reported the list of the 83 most abundant sequences, and their mean concentrations with the standard deviation. The data support the result that the appearance of autocatalytic cycles is not accidental or dependent on a specific choice of initial conditions.

Along with the increase in sequence complexity, new properties can emerge—for example, amino acids bearing carboxylic groups in the side chain permit chelation of metal ions. Another example is the possibility of a disulfide bridge to increase the structural stability. It can be hypothesized that the role of some peptides may be the protection of other peptides from degradation. Finally, when a system is populated with enough copies of each protein, it is easy to imagine the encapsulation of materials in protovesicles. Vesicles can be formed by fatty acids when the concentration of water decreases. Upon water addition, this vesicle begins to swell and splits into two, incorporating a sufficient number of structures to continue the cycle.

It is also important to underline that different sequences correspond to different three-dimensional structures and different protein structures correspond to different functions. In this way, our model offers a framework to accommodate the emergence of new features, their diffusion in the population, the establishment of long-term memory and, above all, the possibility of evolution of the pool of peptides into new and more complex hypercycles. When a peptide becomes long enough, it starts to fold to reach a minimum of free energy. With a specific three-dimensional structure, certain catalytic activity may appear. For example, the appearance of sequences containing 2 or 3 residues of aspartic acid may allow the absorption of metallic cations and this new type of peptide may spread in the population using the mechanism described above.

## 4. Conclusions

Some of the earliest modelings of the origins of life came with the works of Eigen and Kauffman. The authors discussed the interlocking of reaction cycles as an explanation for the self-organization of prebiotic systems. The authors described the so-called hypercycle type of functional organization and stressed its possible relevance concerning the origin and evolution of life. We have shown how the introduction of similarity in the process of mating and degradation of nascent peptides can lead to a significant accumulation of some sequences.

More importantly, it was observed that the small number of sequences together with the concept of similarity allowed the emergence of a kind of dynamic memory. The presence of hypercycles is responsible for the conservation of information over time. The memory of the sequences is not preserved in other molecules such as DNA or RNA but it is an emerging property, and an obvious consequence of the similarity applied to the synthesis. It does not elude our attention that this type of memory can be present in every oscillating system, for example, in the brain. A prebiotic environment in which the presence of catalytic hypercycles may have preceded nucleic acid synthesis is plausible and even likely. It is worth noting that the described model does not explicitly take into account the chirality of amino acids. However, for the above considerations, the similarity may be responsible for a kind of template effect and lead to the accumulation of homochiral sequences. 

This work has thermodynamic sense in an open system. Qualitatively, one can think of entropy S as a “fluid” that can be created or produced and which flows. Stationary states correspond to processes with no variation of entropy. The entropy change is the sum of the entropy changes generated inside the system with those of the entropy exchanged with its surroundings. An open system may undergo a decrease in its symmetry as in the presented work, thanks to the increase of the surrounding environmental entropy. Stability has its thermodynamic expression in Prigogine’s theorem on minimum entropy production [32].

The entropy production reveals the rate of dissipation and the entropy exchange is the flow of entropy to or from the environment. The matter fluxes that drive entropy production are crucial for determining the thermodynamic stability of an open system. The entropy production is an important concept that is central to the thermodynamics of evolution [33]. Even though the investigation of entropy production is of extreme importance, it is not the goal of the present work. Finally, this sort of dynamic memory allowing the accumulation of a continuously expanding set of peptides is the prerequisite to proceeding with the slow optimization of the first proteins through a well-known model of Darwinian evolution. A prebiotic environment in which the presence of catalytic hypercycles may have preceded nucleic acid synthesis is plausible and even likely. 

## Figures and Tables

**Table 1:** The interaction coefficients.

Length	Interaction Coefficient
1	0.083
2	0.168
3	0.310
4	0.500
5	0.690
6	0.832
7	0.917
8	0.968
9	0.982
10	0.992

**Table 2:** The amino acids’ similarity coefficients were calculated by their polarity and ΔG of solvation.

Amino Acid	Similarity Coefficient	Amino Acid	Similarity Coefficient
L	0	M	0.4
I	0	Y	0.6
F	0.1	C	0.6
W	0.2	A	0.7
V	0.4	T	2
G	2.5	H	−6
S	3	R	−6
Q	5	D	8
N	−8	K	−8
E	−9	P	12

**Table 3:** Sequences belonging to the same hypercycle.

Abbreviation	Sequences of the Cluster
α	AAGGAGGGGGGGGGGGGGAGGGAAGGGAAVGGGGG
β	EGAAGGGGGGAGGGGVGGGVAGGGGDGGAGGGGAGAGGGA
χ	GAAEGGVGGDGVAGGGGVGGVAAGAGAGGAGGAVAGGG
δ	AAGGAGAGAGGGGGVGGAGGGVGGAGAGGGGGAGGGG
ε	AGGAAGGEAGGGGAEGEAGAGAGG
φ	GEGEAGAGAGGGAAAGGGGAAGAGVADGGGGAGAAGAGGGAVDGVGAAGGGGG
γ	GEAGGSGAAGGGGGAVGGGGVEGGGEGGGGGGGGGGAGGGG

## Supplementary Materials

The following are available online at https://www.mdpi.com/2075-1729/9/2/33/s1. The program Genesys is freely available upon request to the authors.
