# Supplementary material for: Plausible Emergence of Autocatalytic Cycles under Prebiotic Conditions
Source: Life (Basel). 2019 Apr 4;9(2):33. doi: 10.3390/life9020033 (PMC6617172; doi:10.3390/life9020033)
Supplement: Supplementary file 1 [file life-09-00033-s001.pdf]

Supplementary Information

Table 1 - The table shows the sequences obtained in 50 experiments with slightly different initial conditions varying the concentration of glycine in the initial pool. The pool was constituted by the main 6 amino acids with constant concentration of 38 A, 4 D, 11 E, 100 G, 1 S, and 10 V. The number of glycine spans from 75 to 124. Here are shown the average of the sequence occurrences and their standard deviation. Larger standard deviations are typical in sequences undergoing large fluctuation in different epochs (like periodic oscillation). In bold are shown the largest values.

SEQUENCE	AVERAGE	ST.DEV
AAGGAGAGAGGGGGVGGAGGGVGGAGAGGGGGAGGGG	0.25	0.79
AAGGAGGDGVGGGGGGGGGVGADAGGGVGAEGVAV	0.15	0.49
AAGGAGGGGGGGGGGGGGAGGGAAGGGAAVGGGGG	**0.35**	**1.19**
ADGGGGGGAGVGGGGDVGGGGGGGGG	0.05	0.22
AGAAAAAGGAGGDGGGGGGGGGGGGGGAGGGGGAAAG	0.25	0.79
AGAGVGGGGGGAGVGVGAGVGGVEGGGAGGGAAGGGAAGVGGGGGGEAGGGG	0.10	0.31
AGEGEAGAGAGGGAAAGGGGAAGAGVADGGGGAGAAGAGGGAVDGVGAAGGGGG	**0.45**	**1.39**
AGGAAGGEAGGGGAEGEAGAGAGG	0.10	0.31
AGGAGGAGGAGGEEGGAGGAVGG	0.25	0.55
AGGGADGGGDVGGGGGGG	**0.45**	0.69
AGGGGGGDAAEGGGGGAGSGGGGAAGAGA	0.20	0.52
AGGGVGGGGGVAAGAGVGAGG	0.30	0.57
AGGVAGGAAGEGGAGGAGGGGGA	0.15	0.37
ASGGGGVGAAGGGAGGGGVGGAVGGG	0.20	0.41
DAAGVGAAAGAVAGGGAGGAGEGGGA	0.15	0.49
EGAAGGGGGGAGGGGVGGGVAGGGGDGGAGGGGAGAGGGA	**0.40**	**1.23**
GAAEGGVGGDGVAGGGGVGGVAAGAGAGGAGGAVAGGG	0.10	0.31
GAAGAGAASGAVGAGGAGG	0.15	0.37
GAAGDGAGGGGGGGAGEGAAGGGAGGGGG	0.30	0.98
GAGAVGEGGGGGAGGGAGGGGGDGAA	0.15	0.67
GAGDDGGVAVGGGVGGAA	0.20	0.52
GAGGDGAGAGAGSAGGGGAGG	0.20	0.52
GAGGGAGDAAGGAEAAGAGG	0.15	0.37
GAGGGGGGGGAAGAGGGAGGGGGGGVDGV	0.15	0.49
GAGGGGVAAGGAGEGGAGGGAGGGEVVA	0.05	0.22
GAGGGGVGAAGVGGAGGAEGAGAGGAGGGGADGAGGGGVGGGAGAAGAG	0.20	0.70
GDGGAAGGAGGGVAGGGAAGGA	0.35	0.88
GEAGGSGAAGGGGGAVGGGGVEGGGEGGGGGGGGGGAGGGG	0.25	0.79
GEGAGAGGGGGGGGGAGGGGGGGGVGGGGGAAGGGGAGGVGGG	0.15	0.67
GEGGGGGGAEGGGAGGGGGAGGGGGGGGVGAGGGGGG	0.25	0.79
GGAAGGVGGGGAGGAGGVAD	0.25	0.64
GGAGAAVGGGGVGGAAGGGGGGGGG	0.20	0.31
GGAGGAAGGEAGAGGAGGGGGGVGEGG	0.30	0.98
GGAGGDGAGGEGAAVGEGAGGEAAG	0.10	0.31
GGEDGGGGSGGVGGAGGGG	0.20	0.41
GGEGGAGGAGAGGGAGGGAAGGVGGAGGGVGAAGGGVGGGEA	0.15	0.49
GGGAAGAAAGGGGGGGGGGGGGGGGGGEVGGGGGGGAGAGD	0.15	0.49

GGGAAGDGGGGDGGGGGGGGVAGG	0.30	0.91
GGGDGAAAGGVGGGGGGEAA	0.15	0.37
GGGGGAGGAGAGEEGGGAGVGAGGAGAGGAGGAGGAGAGEGGGEAEGGA	0.10	0.31
GGGGGAGGAGGVGGGGAGGGVAGGGAGGGAGG	0.15	0.49
GGGGGGAGAGGGGAGGGGGG	**0.95**	**1.15**
GGGGGGEDGAGGAGGVAAAGAGGG	0.15	0.37
GGGGGGGAAGAAAAGAGGAA	0.40	0.60
GGGGGGGAGAGGEAGAGGAGAGAAVGGA	0.25	0.72
GGGGGGGGAGSGGGGGAGGGGAGGAGG	**0.50**	**0.95**
GGGGGGGGGGGGGEVVGGGGGAGG	**0.50**	**1.00**
GGGGGGGVAAGGAGAVEAAGGVGGGVGGGAVGGA	0.10	0.31
GGGVGGGGGGAVVAGAGGGA	0.40	0.99
GVGGGEGGEGAGGGGEADGAGV	0.20	0.70
GGGGGGGGSGVGGEEGGGGVAGGAGGGGGAGAGGGGGDGGGGGG	0.10	0.31
AAAGGGGGAGAGGGGAGGEGGGVGGGG	0.10	0.31
AEGGVGGAGGGGAAGAAGAAEG	0.05	0.22
AGGAGGAAAGGGGAAGAGAGAAGGGGDGGGGGGAA	0.05	0.22
AGGGGGGGGEGGGEGDGGGGGGGGGGGAGGGAGGGG	0.05	0.22
AGGGGGGGGGAGGAGAGAVGGAGGGGGG	**0.55**	**1.43**
EAAGAAGGGAGGEGAGGGGGGGGGGGAGEEGDVGAGGG	0.15	0.49
ASAGVGGGGGGGGGEGVAGGVGAGGAGG	0.15	0.49
GGGAGAGGGGEEGGGGEGGGGGSGGAGGGGG	0.15	0.67
GGGAGGGGGGGGVAGGGGGGGGGGGGGGAGGAAAG	0.20	0.67
GGGGAGAAAGAVGGAGAAGGGAAG	0.05	0.22
GGGGEGVGGAGEGEDGGVAVGGDGDAGGGGGGGGVGAGA	0.10	0.31
GVAGGGGGAAGGAVGGEGVGAAGGAAAG	0.05	0.22
VGGAAGGGGGGGAAGGGGGGVVGAGGGAAGGGGG	0.10	0.45
GDGGGAGGGGAGEGGEAGAAGAGGGGGGGGGGGAGG	0.20	0.70
AEGGVGGAGGGGAAGAAGAAEG	0.10	0.31
AGEGGGGGAGGAGGGGGGAGGGVGGGGGAGGGGGGGAGGGGGGGAAEVAAAGEGAGEGGGGDV	**0.35**	**1.09**
AGGAGGDGGGAAGDDGGG	0.05	0.22
AGGGGGGGGGAGGAGAGAVGGAGGGGGG	0.15	0.49
DGGGAGVEGAGAGGGAAVGAGAGGGGVGG	0.05	0.22
GAEEGAGAAGGAAGAAGGGAAAGG	0.05	0.22
GGAGAGGGAAAVGGEGAAADGAE	0.05	0.22
GGGAGAGGGGEEGGGGEGGGGGSGGAGGGGG	0.10	0.45
GGGAGGGGGAAAGAGGGGGEGADGAGGAGGAGDGAGA	0.05	0.22
GGGAGGGGGGGGVAGGGGGGGGGGGGGGAGGAAAG	**0.35**	**1.09**
GGGEAGGGGAGGVGAGGAGVGGGGGGEVGAGGGGAGGAAA	0.10	0.31
GGGGAGAAAGAVGGAGAAGGGAAG	0.05	0.22
VGGGGGGVGGGGGGAAGGGGGAGGAGGGEGGGEGVGDG	0.10	0.45
GGADEGGGGGGGGDGVAAGGVGDGGGGGG	0.15	0.67
GGAGAAGVGGGVGGAGGGVGGGGGGGAGGGGGAAGGGG	0.15	0.67
